# Narcissistic traits and compassion: Embracing oneself while devoiding others

**DOI:** 10.3389/fpsyg.2022.914270

**Published:** 2022-10-11

**Authors:** Vanessa Lea Freund, Frenk Peeters, Cor Meesters, Nicole Geschwind, Lotte H. J. M. Lemmens, David P. Bernstein, Jill Lobbestael

**Affiliations:** Department of Clinical Psychological Science, Faculty of Psychology and Neuroscience, Maastricht University, Maastricht, Netherlands

**Keywords:** grandiose narcissism, vulnerable narcissism, self-compassion, other-compassion, induction

## Abstract

Grandiose narcissistic traits refer to exploitative and arrogant attitudes, while vulnerable narcissistic traits entail hypersensitivity to judgment and low self-esteem. Little is known about how individuals with narcissistic traits can improve their attitudes toward themselves and others. The current research puts self- and other compassion forward as possible targets to alleviate some of destructive patterns of narcissism. Generally, self-compassion (SC) has previously been associated with beneficial effects on psychological wellbeing, while other compassion (OC) is advantageous for interpersonal relationships. This study explored the relationship between narcissistic traits and the efficacy of experimental compassion inductions. Student and community participants (*N* = 230, *M_*age*_* = 27.41, 65.2% female) completed grandiose and vulnerable narcissistic trait, SC and OC state questionnaires, and either an SC or OC induction. It was expected that individuals with higher narcissistic traits (particularly grandiose traits) would benefit from the inductions and show higher SC after but would have greater difficulty showing meaningful increases in OC (especially OC directed at the general population). The results indicated that individual differences in grandiose and vulnerable narcissistic traits are related to the magnitude of improvements following the inductions: the theorized lack of SC in individuals with vulnerable oversensitivity to judgment traits seems possible to be counteracted through different types of compassion exercises. Moreover, higher grandiose exploitativeness–entitlement and global vulnerable narcissistic traits related to less increases than others. However, directly inducing OC in individuals with these traits was linked to greater OC improvements than improvements after inducing SC. Overall, the present findings suggest that self-compassionate behavior can be improved in individuals with high oversensitivity and that other compassionate behavior could potentially be increased if, specifically, other compassion exercises are utilized when higher levels of certain narcissistic traits are present.

## Introduction

Research into grandiose and vulnerable narcissism has been conducted steadily; however, not much is known about improving the wellbeing or the interpersonal relationships of individuals who exhibit narcissistic traits in the long run. The core of narcissism entails a cognitive-affective pre-occupation with the self, with an observable tendency to put one’s needs above everyone else (Rhodewalt and Morf, 1998), and includes a highly agentic self-view (e.g., Campbell et al., 2006). While within the concept of narcissism, multiple conceptualisations exist (e.g., Miller et al., 2021), we will focus on two factors, grandiosity and vulnerability. Grandiose narcissism is characterized by superiority, exploitativeness, grandiosity, and arrogance (Pincus et al., 2014). Vulnerable narcissism entails hypersensitivity to judgment, neuroticism (Miller et al., 2018), and low self-esteem (Rogoza et al., 2018). Individuals with vulnerable narcissistic traits depend on external validation and tend to interpret their environment negatively through hostile attribution biases (Zeigler-Hill et al., 2008; Miller et al., 2011). Long-term grandiose and vulnerable narcissistic traits can be devastating to both interpersonal relationships and the self. For example, narcissism is related to relationship issues. Specifically, grandiose narcissism is linked to narcissistic rivalry, that is, derogating others (Wurst et al., 2017) or using relationships to self-regulate emotions and behaviors to support an agentic self-view (e.g., Campbell et al., 2002), while vulnerable traits are related to increased victim mentality and partner mistreatment (Brown, 2017). Adverse effects on the self of both types of narcissism include increased non-suicidal self-harm (Dawood et al., 2018), and vulnerable narcissism specifically relates to reduced life satisfaction and increased anxiety, depression, and suicidal ideation (Miller et al., 2011; Rohmann et al., 2019; Ponzoni et al., 2021). As such, it begs the question if there are attributes that can alleviate the strain put on the self and others posed by individuals with grandiose and vulnerable narcissistic traits.

Both sets of narcissistic traits have been associated with therapy resistance (Kernberg, 2007). However, it was observed that grandiose narcissistic behaviors improved after single inductions when an increase in a communal focus was targeted (Konrath et al., 2006; Finkel et al., 2009; Giacomin and Jordan, 2014; Jordan et al., 2014). Specifically, when a sense of personal connection, that is, a focus on others, was induced by telling participants that they share a birthday, narcissistic aggression reduced in the moment (Konrath et al., 2006), and priming communal thoughts led to a higher momentary commitment to the narcissists’ partner (Finkel et al., 2009). Moreover, while narcissism is related to reduced empathy for others, it seems that this does not reflect an inability to be empathic. To this end, when individuals higher in grandiose narcissistic traits were instructed to take another person’s perspective before watching a video of a person in distress, they reported higher empathy levels post-video (Hepper et al., 2014). Furthermore, in response to an emotionally distressing video, individuals with higher maladaptive narcissistic traits did show a lower heart rate than controls; however, when instructed to take the distressed person’s perspective before watching the video, the heart rate did not decline. This shows that pro-sociality, which tends to be negatively associated with narcissism, may be adaptive and alludes to its potential to be induced and trained. Similarly, another behavior that could be therapeutically targeted to address narcissistic behavior is compassion; previous observations showed that narcissistic traits were associated with low self- and other compassion (Barry et al., 2015; Salazar, 2016; Brouns et al., 2020).

Self-compassion (SC) is an adaptive emotion regulation strategy and attitude toward oneself that entails recognizing hardship on an emotional, cognitive, and attention level and confronting it with kindness, common humanity, and mindfulness (Neff, 2003a; Elices et al., 2017). SC incorporates trait elements that seem to predispose individuals to be apt to employ self-compassionate attitudes (Waring and Kelly, 2019), in addition to state elements that are acquirable and adaptable through inducing and training SC (Leary et al., 2007; Dodson and Heng, 2022).

The SC has been associated with beneficial effects on mental health, such as lower anxiety (Neff et al., 2007), burnout (Dev et al., 2020), and aggression (Barry et al., 2015), and increased motivation to self-improve (Breines and Chen, 2012). Meanwhile, other compassion (OC) is seen as a pro-social motivation that involves recognizing hardship in others while wanting to help and alleviate it (Kirby et al., 2019). OC comprises components that parallel self-compassion, that is, kindness, common humanity, and mindfulness (Pommier et al., 2019), which can be directed at a specific individual that is known or at not one person but the general population (i.e., specific or general OC, see Freund et al., under review^1^). OC was previously associated with increased pro-social behavior, such as better patient care in healthcare settings (Conversano et al., 2020), increased activation of brain regions associated with reward and affiliation (Klimecki et al., 2014), and reduced interpersonal conflict levels (Scarlet et al., 2017). Thus, increasing SC and OC in individuals with grandiose and vulnerable narcissistic traits could prove to be a desirable tool to reduce their adverse effects.

### Grandiose narcissistic traits and compassion

Even though grandiose narcissistic traits are related to greater subjective wellbeing (Sedikides et al., 2004; Kaufman et al., 2020) and seem to portray a self-confident persona that is generally associated with high self-esteem (e.g., Rohmann et al., 2012), this may not be the complete picture. It is thought that grandiose individuals, in fact, employ this persona as a defense strategy and that it may, in reality, mask an easily threatened ego or a reactive decrease in self-esteem (Raskin et al., 1991; Zeigler-Hill et al., 2010). Consequently, it would be expected that this fragility would be mirrored in low SC. Indeed, with one exception that found no significant relationship (Neff, 2003b), previous research supports this expectation. Specifically, grandiose narcissistic traits were significantly related to reduced SC (Barnett and Flores, 2016; Demirci et al., 2019) or were weakly but not significantly related to lower SC (Barry et al., 2015). As a result, it seems that individuals with grandiose narcissistic traits could benefit from increasing their SC to mitigate their fragile egos.

Interpersonally, grandiose narcissistic relationships tend to be defined by low commitment, high stress, and conflicts (e.g., Campbell and Foster, 2002; Campbell et al., 2011). Individuals with grandiose narcissistic traits reported choosing relationships based on the partner’s intelligence, their ability to make them feel good, and attractiveness (Jonason and Schmitt, 2012). They were also more insensitive to the importance of those close to them and more likely to report negative perceptions of them (Lamkin et al., 2014). This portrays a superficial and self-centered focus on relationships, which hints at a lack of deeper feelings for others, such as a deficiency in OC. Unsurprisingly, grandiose narcissistic traits were indeed previously related to low compassionate love (Brouns et al., 2020). Furthermore, a meta-analytic review found an overarching relation to self-reported reduced cognitive and affective empathy (Urbonaviciute and Hepper, 2020), which is strongly related to OC and may be required to elicit compassionate responses (e.g., Stevens and Woodruff, 2018). Consequently, this renders OC a desirable trait for those high in grandiose narcissistic traits and leaves room to explore the feasibility of increasing OC in these individuals.

### Vulnerable narcissistic traits and compassion

Vulnerable narcissistic traits were previously reported to relate to adverse effects on psychological health, such as increased self-alienation and guilt, reduced life satisfaction, self-acceptance, motivation at work, psychological wellbeing, and self-esteem (Sedikides et al., 2004; Neff and Vonk, 2009; Zessin et al., 2015; Kaufman et al., 2020; Wirtz and Rigotti, 2020). Furthermore, vulnerable narcissistic traits have been shown to strongly relate to low SC (Barry et al., 2015; Gu and Hyun, 2021), which hints at the assumption that increasing SC could also benefit those with vulnerable narcissistic traits.

On an interpersonal level, the relationship between vulnerable narcissistic traits and OC has not been investigated directly; however, research suggests an association between vulnerable narcissistic traits and reduced OC, as measured by proxies and closely related constructs. Vulnerable narcissistic traits have been negatively related to perspective taking, empathic concern, emotional intelligence, theory of mind, and adaptive cognitive and affective empathy (Vonk et al., 2013; Aradhye and Vonk, 2014; Luchner and Tantleff-Dunn, 2016; Urbonaviciute and Hepper, 2020). Additionally, vulnerable narcissistic traits have been associated with a variety of measures indicating lower relationship quality. As such, individuals with vulnerable narcissistic traits reported fewer positive relationships (Kaufman et al., 2020). Moreover, although less strongly than grandiose narcissistic traits, those with elevated vulnerable narcissistic traits also reported a negative perception of their close circle (Lamkin et al., 2014). Furthermore, vulnerable narcissistic individuals reported higher interpersonal distress, more cold and socially avoidant interpersonal problems (Dickinson and Pincus, 2003), and higher attachment anxiety (Rohmann et al., 2012), thus leaving the question if targeting this problematic interpersonal relationship style by increasing compassion for others may result in advantageous outcomes.

### The present study

We recently conducted research in which we experimentally induced compassion through SC or OC writing tasks while measuring trait and state SC and OC. In a previous study, SC and OC were both malleable and did not only increase their corresponding concept through direct, specific effects but also spilled over to other concepts (i.e., inducing SC also increased OC, and vice versa) [Freund et al., under review (see footnote 1)]. We now aimed to assess the relationship between grandiose and vulnerable narcissistic traits, and the induced changes in state SC and OC. The results could provide insights into possible intervention strategies for narcissism. Overall, the general pattern we expected was that individuals high in narcissistic traits (particularly grandiose traits) would increase their SC more easily than individuals who scored lower in narcissistic traits but would have greater difficulty increasing their OC (especially general OC).

Given the self-centered focus of grandiose narcissistic traits (e.g., Rhodewalt and Morf, 1998; Pincus et al., 2014), it was expected that this narcissistic pre-occupation with the self could be utilized during the inductions to gain greater self-serving SC. Thus, grandiose narcissistic traits were expected to be positively related to improvements in SC. Regarding the superficial, high-conflict relationships individuals with elevated grandiose narcissistic traits tend to have (e.g., Campbell et al., 2006; Wurst et al., 2017) and given their negative association with pro-social attributes (e.g., Urbonaviciute and Hepper, 2020), we expected that grandiose narcissistic traits will also negatively relate to improvements in OC. However, narcissism is further related to the narcissistic use of relationships to self-regulate an agentic self-view (Campbell et al., 2006), the protection of relevant close targets (Leibowitz, 1997), and in-group members in association with collective narcissism (Cichocka and Cislak, 2020), which entails an unrealistic, positive view on one’s in-group members and over-identifying with them (e.g., Żemojtel-Piotrowska et al., 2021). This collectivism serves as an enhancement of the self, which is egocentricity-driven, rather than for the benefit of the group (e.g., Cichocka and Cislak, 2020). Given that this extension of the self might also be employed for OC, grandiose narcissistic traits were expected to improve OC directed toward an individual close to oneself (i.e., specific OC) moderately more than OC directed toward the general population (i.e., general OC) and thus individuals who do not benefit the narcissistic individual.

**FIGURE 1:**
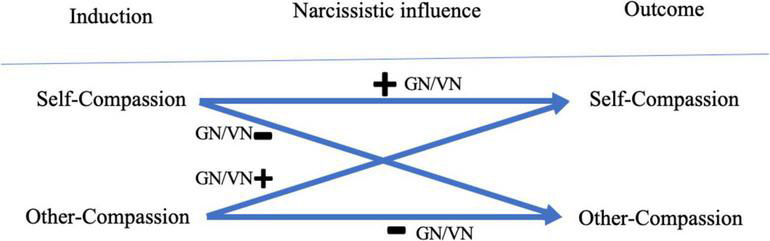
Visualization of the hypotheses. +, predicts a positive relation to an increase in compassion. –, predicts a negative relation to an increase in compassion.

Consequently, based on the self-absorbed and interpersonally challenging nature of grandiose narcissistic traits, the following hypotheses were proposed (Figure 1):

*Hypothesis i: Grandiose narcissistic traits will be positively related to an increase in SC following the inductions. This relation will be more prominent after the SC than after the OC induction, thus individuals with higher grandiose narcissistic traits will improve their SC, especially after the SC compared to the OC induction.*

*Hypothesis ii: Grandiose narcissistic traits will be negatively related to an increase in OC following the inductions. This relation will be more prominent after the OC than after the SC induction. This will be more prominent for general OC than specific OC, thus individuals with higher grandiose narcissistic traits will not show meaningful improvements in OC, especially after the SC induction, and most noticeably regarding general OC.*

Moreover, while vulnerable narcissistic traits previously were related to reduced positive intrapersonal wellbeing and interpersonal behavior, similar hypotheses to the grandiose narcissistic hypotheses were expected. This was presumed based on their shared sense of self-centeredness and self-absorption, which may enable high scorers to immerse themselves pre-eminently into the inductions to acquire SC and on their association with various indicators for interpersonal relationship difficulties, such as reporting fewer positive relationships (Kaufman et al., 2020) or cold and socially avoidant behaviors (Dickinson and Pincus, 2003), which may potentially interfere with inducing OC. However, considering the insecure nature of vulnerable narcissistic traits, in which outside validation is sought (Crocker, 2002), it was anticipated that the effects would be weaker than grandiose narcissistic traits. This resulted in the following hypotheses (Figure 1):

*Hypothesis iii: Vulnerable narcissistic traits will be positively related to an increase in SC following the inductions, although less pronounced than grandiose narcissistic traits. This relation is more prominent after the SC than after the OC induction, thus individuals with higher vulnerable narcissistic traits will improve their SC, especially after the SC than after the OC induction.*

*Hypothesis iv: Vulnerable narcissistic traits will be negatively related to an increase in OC following the inductions, although less pronounced than grandiose narcissistic traits. This relation will be more prominent for general OC than specific OC and stronger after the OC than the SC induction, thus individuals with higher vulnerable narcissistic traits will not show meaningful improvements in OC, especially after the SC induction, and most noticeably regarding general OC.*

Lastly, considering that different components constitute the overall narcissistic picture, and there is currently no knowledge as to which specific narcissistic characteristics relate to compassion, the subscale scores for grandiose and vulnerable narcissistic traits will be exploratively analyzed individually in relation to the aforementioned hypotheses. As such, grandiose narcissistic traits are divided into four subscale scores of leadership–authority, self-absorption–self-admiration, superiority–arrogance, and exploitativeness–entitlement (Emmons, 1987). Their individual contributions are important to consider. For example, leadership–authority is viewed as the most adaptive most adaptive of the grandiose presentations (Cai and Luo, 2018) and may consequently be beneficial while increasing SC. Conversely, exploitativeness–entitlement is the most maladaptive and devastating to interpersonal relationships (Cai and Luo, 2018) and may thus play a greater role in hindering the acquisition of OC. Similarly, vulnerable narcissistic traits can be divided into two subscales: oversensitivity to judgment and egocentrism (Fossati et al., 2009). The former represents a more vulnerable self, which may play a role in SC. By contrast, the latter represents the narcissistic core, which presumably may interact and stifle the acquisition of OC. Since each subscale provides unique factors, their particular contribution may shed light on each impact on SC and OC.

## Materials and methods

The data were collected as part of a larger research project on the malleability of compassion [see Freund et al., under review (see footnote 1); for pre-registration^2^, data, and syntax^3^, ^4^]. For this study, only materials, procedures, and analyses^5^ relevant to the current research questions are described.

### Participants

The sample consisted of *N* = 230 participants (female 65.2%), with a mean age of 27.41 (*SD* = 10.85; range 18–65) years, after exclusion based on the attention checks and the adherence to the task instructions of the inductions from an original sample of *N* = 273. About 32% of participants were German, 28.7% Dutch, 8.7% Belgian, and 30.4% were of 27 other nationalities. The majority of the highest completed education was high school (37.4%) and university (32.2% Bachelor; 19.1% Master). The sample consisted of 58.3% students, 28.7% employed, and 13% otherwise engaged. Most participants were in a relationship or married (51.3%) or single (47.4%). The rest was either divorced or did not specify their relationship status. The majority (96.5%) of our sample did not take psychoactive medication. Although the research was available in Dutch and English, most participated in English (71.3%).

### Materials

The measures used were administered in their validated English or Dutch versions. If the latter was not available (state self-compassion scale; state general and specific other compassion scale; inductions), translations were made by the researchers by independently translating and back-translating to achieve a valid translation.

#### Grandiose narcissism

The 37-item Narcissistic Personality Inventory (NPI; Raskin and Hall, 1979; Morf and Rhodewalt, 1993; Barelds and Dijkstra, 2010) was used to measure grandiose narcissistic traits. Only items with factor loadings greater than 0.35 are employed in this NPI version (Emmons, 1987; Brown and Zeigler-Hill, 2004). Each item was rated on a seven-point Likert scale ranging from 1 (*strongly disagree*) to 7 (*strongly agree*). The NPI has four subscales: leadership–authority (LA), self-absorption–self-administration (SA), superiority–arrogance (SU), and exploitativeness–entitlement (EX) (Emmons, 1987). The NPI has a high test–retest reliability, *r* = 0.81 (del Rosario and White, 2005); adequate to excellent internal consistencies, α = 0.68–0.87 (Emmons, 1987; Brown and Zeigler-Hill, 2004); and good construct validity, *r* = 0.37–0.71 (Raskin and Terry, 1988). McDonald’s omegas for the current sample were ω = 0.90, 80, 82, 76, and 76 for the global score, LA, SA, SU, and EX, respectively.

#### Vulnerable narcissism

The 10-item Hypersensitive Narcissism Scale (HSNS; Hendin and Cheek, 1997; de Bruin et al., 2017) was used to measure vulnerable narcissistic traits. Items were rated on a seven-point Likert scale ranging from 1 (*strongly disagree*) to 7 (*strongly agree*). The HSNS has a two-factor structure: oversensitivity to judgment (OJ) and egocentrism (EC) (Fossati et al., 2009). It has good test–retest reliability (*r* = 0.63); adequate internal consistencies of α = 0.66 for OJ, 0.62 for EC, and 0.71 for the global scores (Fossati et al., 2009); and good construct validity (Hendin and Cheek, 1997). McDonald’s omegas for the current sample were ω = 0.74, 69, and 0.62 for the global score, OJ, and EGO, respectively.

#### State self-compassion

The state Self-Compassion Scale (Neff et al., 2020) was used, in which the participants were asked to think of a painful or difficult situation in their life while answering the questions. The scale consists of 18 items scored on a five-point Likert scale ranging from 1 (not at all true for me) to 5 (very true for me). Internal consistency of the total score was previously found to be between α = 0.88 and 0.94, and its composite reliability between *CR* = 0.93 and 0.97 in a student and a community sample (Neff et al., 2020). McDonald’s omega in the current sample was ω = 0.91 (pre-induction) and 0.90 (post-induction).

#### State other compassion (general)

The authors developed the general state other compassion scale by adapting the Compassion Scale items (Pommier et al., 2019) to match the language in the state Self-Compassion Scale (see Appendix 1). The questionnaire asked the participants to indicate how they feel about people in general (everyone, strangers, neighbors, etc.) at the current moment. The final version included 16 items, scored on a five-point Likert scale ranging from 1 (*not at all*) to 5 (*very true for me*). Internal consistencies were acceptable, with α = 0.70 for the pre-measure and 0.74 for the post-measure. McDonald’s omega in the current sample was ω = 0.86 (pre-induction) and 0.91 (post-induction).

#### State other compassion (specific)

The authors generated the state-specific other compassion scale by adapting the Compassion Scale items (Pommier et al., 2019) to match the language in the state Self-Compassion Scale while consulting with Kristin Neff, who developed previous (self)compassion scales (see Appendix 2). The instructions asked the participants to think about a difficult or painful situation someone they know (e.g., a family member or a friend) is experiencing while answering the items. It includes 16 items, which were scored on a five-point Likert scale ranging from 1 (*not at all*) to 5 (*very true for me*). Internal consistencies were acceptable with α = 0.70 for both the pre- and post-measures. McDonald’s omega in the current sample was ω = 0.84 (pre-induction) and 0.85 (post-induction).

#### Self-compassion induction

The Self-Compassionate Mindset Induction (Neff et al., 2020) was used. The participants were asked to think of a specific situation that is currently painful or difficult for them. The induction is composed of three positive components of SC: mindfulness, common humanity, and kindness. Each of the three writing prompts encouraged the participants to write at least 200 words. The writing task was followed by an manipulation check that asked the participants to select one correct option, of three, as to what the writing task asked of them.

#### Other-compassion induction

OC was facilitated using the Other-compassionate mindstate induction, which the current authors adapted to mirror Neff et al. (2020) SC mindstate induction in consultation with Kristin Neff (see Appendix 3). The wording of the SC induction was changed to facilitate OC by instructing the participants to think of a situation that is currently painful or difficult for someone they know while asking them to relate to their hardship. The induction followed the three positive components of OC: mindfulness, common humanity, and kindness. Each writing prompt instructed the participants to write at least 200 words addressed to their chosen person; 52.2% chose a friend, 13.5% a parent, and 7.4% a grandparent. The rest chose either siblings, distant relatives, or colleagues. The last step included an attention check similar to the one in the SC induction.

### Procedure

Initially, the participants were recruited at Maastricht University through flyer advertisement and invited to individual 1-h timeslots. After the first 10 participants, recruitment and testing moved online due to COVID-19 restrictions. The participants were stratified based on their sex and student/general population status to ensure even distribution between these two inductions. The participants were briefed and gave written consent. After, the participants completed the demographical data and the trait measures. Depending on the stratification, the subsequent instructions followed the SC induction (*N* = 115) or the OC induction (*N* = 115). Next, the participants continued to fill out the pre-induction state measures and the induction. Once the writing task was complete, the participants were asked to reread their text carefully before answering the manipulation check, which asked the participants to explain the nature of the induction. After that, they completed the post-induction state measures. Throughout the tasks, the participants completed attention checks at multiple points, in which they had to confirm the content of the previous instructions they had received. Upon completion, the participants were debriefed and received either university participation credits or a voucher worth €12.50. The Ethical Review Committee of Psychology and Neuroscience of Maastricht University approved the research (218_11_02_2020).

### Data preparation

A sample size calculation was performed for multiple linear regression; *r*^2^ increase (G*power, Faul et al., 2009), with an effect size *f*^2^ of 0.05, alpha error probability of 0.05, power of 0.9, and eight predictors (grandiose and vulnerable narcissistic scores and subscales). The calculation determined a total sample size of 213 participants.

The participants were included in the final sample if they passed the attention checks, were oblivious to the research aim, and understood the induction writing task. A total of two independent raters assessed the writing task based on the participants’ adherence to the instructions, in which the raters coded the induction outcomes as ‘include,’ ‘exclude,’ or ‘equivocal.’ In equivocal or disagreement cases, a third rater was consulted. Of the initial 273 participants, 33 were excluded because of this. The inter-rater reliability of the two independent raters (2,k ICC for absolute agreement) was excellent, *r* = 0.844 (Cicchetti, 1994). Additionally, 10 participants were excluded due to failing the attention checks (final sample = 230). McDonald’s omegas for the scales were calculated using the SPSS extension ‘Omega, Alpha, and all Subsets Reliability Procedure’ *version 1.0* by Hayes (Hayes and Coutts, 2020).

### Statistical analyses

To test for baseline differences between the average trait and compassion pre-induction scores of both induction groups, independent samples *t*-tests were completed. To give an indication on whether the inductions were successful at increasing compassion in general, dependant *t*-tests were conducted to test if there were significant differences between pre- and post-induction compassion scores. Pre- to post-induction changes in compassion scores for each state measure were calculated (post- to pre-measure). Overall, the higher the change score, the stronger the increase in compassion. Furthermore, general and specific OC were added together and divided by 2 to calculate a total OC score. Pearson correlations were used to test the relationship between the study variables.

To test hypotheses i and ii (SC and OC and grandiose traits), the SPSS extension PROCESS macro by Hayes (v.2.5.3; Hayes, 2017) was used, where change scores were used as Y variables, the induction group as the X variable (SC induction coded as 1; OC induction coded as 2), and the grandiose narcissistic traits (global and subscales) as moderator variables W. This resulted in 20 regression moderation analyses, in which five analyses (one for the global grandiose narcissistic trait score, and four for the additional subscales) for each of the four different change scores were conducted. Model number 1 was applied, with heteroscedasticity-consistent inference (HC3), centring for continuous variables, the Johnson–Neyman method, and *R*^2^s were calculated for effect sizes. Probing within the PROCESS macro analyses split the narcissistic traits into low, medium, and high scores based on ±1 standard deviations. PROCESS interaction effects were probed when *p* < 0.05.

Again, to test hypotheses iii and iv (SC and OC and vulnerable traits), PROCESS macro by Hayes was used. The same variables and procedure as above were chosen, only now with vulnerable narcissistic traits as W variables. In total, 12 moderation regression analyses were conducted; for each of the four change scores, three analyses were conducted (global vulnerable narcissistic traits and the two subscales individually).

## Results

Trait, state, and change scores for the final sample and stratified per induction are presented in Table 1. Independent samples *t*-tests were conducted to explore baseline differences between the induction conditions based on trait scores. No significant baseline differences were detected within the trait or compassion pre-scores (see Table 1). Based on face validity, the mean narcissistic trait scores seem comparable with those of other research with similar sample characteristics (e.g., Ng et al., 2014; Lobbestael et al., 2016). See Appendix 4 for Pearson correlations between the study variables.

**TABLE 1:** Descriptive table (mean and SD) and independent samples *t*-tests.

	Mean (std. deviation)			*T*-test	
	Total *N* = 230	SC induction *N* = 115	OC induction *N* = 115	*T*	*P*
**Grandiose narcissism (NPI)**					
Global	3.85 (0.69)	3.82 (0.67)	3.88 (0.71)	−0.72	0.40
Leadership-authority	4.18 (1.02)	4.16 (1.02)	4.19 (1.02)	−0.18	0.95
Self-absorption-self-administration	4.25 (0.89)	4.32 (0.84)	4.18 (0.93)	1.18	0.29
Superiority-arrogance	3.57 (0.78)	3.51 (0.80)	3.62 (0.75)	−1.05	0.90
Exploitativeness-entitlement	3.41 (0.93)	3.27 (0.94)	3.55 (0.91)	−2.30	0.72
**Vulnerable narcissism (HSNS)**					
Global	3.41 (0.81)	3.53 (0.79)	3.73 (0.83)	−1.85	0.90
Oversensitivity to judgment	4.33 (0.99)	4.22 (0.94)	4.45 (1.04)	−1.71	0.45
Egocentrism	2.94 (0.90)	2.86 (0.86)	3.02 (0.92)	−1.28	0.42
**Pre-induction**					
State self-compassion	3.51 (0.74)	3.42 (0.73)	3.59 (0.74)		
**State other-compassion**					
Total	4.15 (0.49)	4.18 (0.50)	4.13 (0.47)		
General	4.03 (0.58)	4.09 (0.58)	3.96 (0.58)		
Specific	4.28 (0.53)	4.26 (0.54)	4.29 (0.52)		
**Post-induction**					
State self-compassion	3.81 (0.64)	3.89 (0.59)	3.73 (0.68)		
**State other-compassion**					
Total	4.28 (0.50)	4.26 (0.52)	4.31 (0.49)		
General	4.16 (0.62)	4.14 (0.62)	4.17 (0.61)		
Specific	4.41 (0.50)	4.37 (0.51)	4.44 (0.49)		
**Change**					
Self-compassion	0.30 (0.52)	0.46 (0.56)	0.14 (0.43)		
**Other-compassion**					
Total	0.13 (0.32)	0.08 (0.30)	0.18 (0.33)		
General	0.13 (0.45)	0.05 (0.41)	0.21 (0.48)		
Specific	0.13 (0.32)	0.11 (0.32)	0.15 (0.32)		

Dependant *t*-tests (Appendix 5) show that post-induction compassion scores were significantly greater than pre-induction scores for all types of compassion. The main effects of the induction type can be seen in the induction column of Appendices 6, 7. Here, the type of induction was significant for SC, total OC, and general OC, but not for specific OC. This implies that the SC induction was significantly more successful at improving SC than the OC induction, as evidenced by the negative value of the main induction effect. Additionally, the OC induction was positively related to significantly greater improvements in total and general OC than the SC induction, as evidenced by the positive value of the main induction effect. Thus, the SC induction was more effective at improving SC, and the OC induction was more effective at improving OC (total and general).

### Grandiose narcissistic traits and the induction of compassion

Hypothesis i (*individuals with higher grandiose narcissistic traits will improve their SC, especially after the SC compared to the OC induction*) was not supported by the main analyses. Grandiose narcissistic traits did not relate positively to an increase in SC after the inductions, and neither did they relate negatively (Appendix 6; model 1–5; trait and interaction column).

**FIGURE 2:**
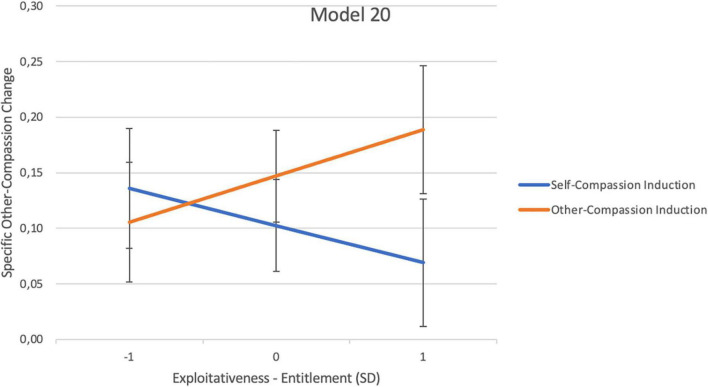
Moderation regression interaction plot; displaying the average specific other-compassion change scores for pick-a-point values (SD) of grandiose exploitativeness-entitlement traits for each induction type. The *y*-axis represents the pre-to-post induction specific OC change scores. Change scores of zero indicate no improvement or worsening. The higher the value on the *y*-axis, the larger the improvement. The error bars represent the standard error.

Hypothesis ii (*individuals with higher grandiose narcissistic traits will not show meaningful improvements in OC, especially after the SC induction, and most noticeably regarding general OC*) was partially supported. The analyses revealed a significant main effect of the grandiose narcissistic traits of exploitativeness–entitlement for total OC with a moderate effect size (Osteen and Bright, 2010) (model 10, Table 2). This indicates that the least improvement in total OC was observed when exploitativeness–entitlement was high, irrespective of whether the induction was aimed at SC or OC. Moreover, with a small effect size, exploitativeness–entitlement significantly interacted with the inductions for specific OC (model 20, Table 2 and Figure 2). The probing revealed that higher exploitativeness–entitlement scores related to significantly greater specific OC increases following the OC induction than the SC induction. However, both inductions still produced increases in specific OC, although with different magnitudes (Figure 2). This interaction plot indicates that higher exploitativeness–entitlement showed the smallest improvements in specific OC after the SC induction and the largest improvements after the OC induction.

**TABLE 2:** Significant moderation regression analyses for grandiose narcissistic traits.

Change	Model	Induction		Trait		Interaction: induction × trait			Effect size
State	#	*t*	*P*	*T*	*P*	Score	*t*	*P*	*R* ^2^
Total other-compassion	10			Exploitativeness-entitlement					
		2.66*	0.009	−2.05*	0.04		1.51	0.13	0.04
	20			Exploitativeness-entitlement					
Specific other-compassion		1.07	0.29	−1.91	0.06		2.02*	0.04	0.02
						Low	−0.57	0.57	
						High	2.08*	0.04	

*p < 0.05.

### Vulnerable narcissistic traits and the induction of compassion

Hypothesis iii (*individuals with higher vulnerable narcissistic traits will improve their SC, especially after the SC compared to the OC induction*) was partially supported (see Appendix 7). The results revealed a significant positive main effect of the vulnerable narcissistic trait “oversensitivity to judgment” for SC change (model 22, Table 3), with a large effect size. This significance indicates that higher oversensitivity to judgment scores related to the greatest increases in SC, irrespective of whether the induction was focused on increasing SC or OC.

**TABLE 3:** Significant moderation regression analyses for vulnerable narcissistic traits.

Change	Model	Induction		Trait		Interaction: induction × trait			Effect size
State	#	*t*	*P*	*t*	*P*	Score	*t*	*P*	*R* ^2^
Self-compassion	22			Oversensitivity to judgment					
		−5.42*	<0.0001	2.33*	0.02		−1.58	0.12	0.15
Total other-compassion	24			Global					
		2.37*	0.02	−2.08*	0.04		2.47*	0.01	0.05
						Low	.09	.93	
						High	2.99*	.003	
	25			Oversensitivity to judgment					
		2.31*	0.02	−1.70	0.09		2.15*	0.03	0.05
						Low	0.38	0.71	
						High	2.90*	0.004	
Specific other-compassion	30			Global					
		0.89	0.38	−1.60	0.11		2.35*	0.02	0.04
						Low	−0.93	0.35	
						High	2.11*	0.04	

*p < 0.05. The significance of the type of induction can be seen in the induction column.

**FIGURE 3:**
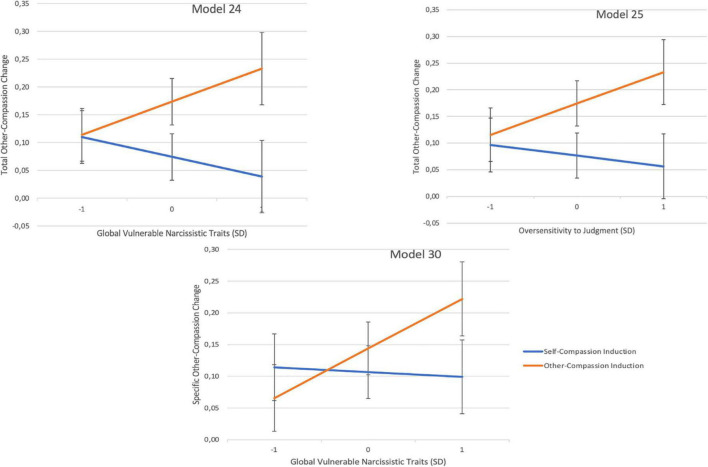
Moderation regression interaction plots; displaying the average other-compassion change scores for pick-a-point values (SD) of vulnerable narcissistic traits for each induction type. The *y*-axis represents the pre-to-post induction OC change scores. Change scores of zero indicate no improvement or worsening. The higher the value on the *y*-axis, the larger the improvement. The error bars represent the standard error.

Furthermore, hypothesis iv (*individuals with higher vulnerable narcissistic traits will not show meaningful improvements in OC, especially after the SC induction, and most noticeably regarding general OC*) was partially supported. Global vulnerable narcissistic traits had a moderate, negative, and significant main effect on total OC change (model 24, Table 3). Hence, higher vulnerable narcissistic traits were associated with the least improvement in OC. Moreover, the interaction effect was significant between the inductions for high global vulnerable narcissistic traits and oversensitivity to judgment for total OC (models 24 and 25; Table 3 and Figure 3). Precisely, higher levels of these traits were related to significantly greater improvements in total OC after the OC induction than after the SC induction.

Lastly, global vulnerable narcissistic traits significantly interacted with the inductions (model 30; Table 3), with a moderate effect size. The probing revealed higher vulnerable narcissistic traits were associated with significantly greater increases in specific OC after the OC induction than after the SC induction (Figure 3).

## Discussion

This research investigated the efficacy of self- and other compassion inductions at increasing state self- and other compassion while taking individual differences in grandiose and vulnerable narcissistic traits into account.

### Grandiose narcissistic traits and compassion

Grandiose narcissistic traits overall did not relate to a decreased nor increased ability to improve compassion for others. Only exploitativeness–entitlement is negatively linked to improvements in OC, irrespective of the induction. However, when OC is directly targeted, it seems that exploitativeness–entitlement is able to increase compassion for close others.

To be precise, the hypothesis that grandiose narcissistic traits would aid in increasing SC after the inductions (hypothesis i) was not supported by the main analyses. While the exact reasons for this are unclear, it may relate to narcissism maladaptive processing of devaluation (Funk, 2018), in which self-worth is dependent on external input and the lack results in scarce self-love (Fromm, 2013; Henschke and Sedlmeier, 2021). Following this line of reasoning, it seems that without the external input, there may be an inherent inability to solace the self and internalize compassion in the absence of extrinsic guidance and validation.

The hypothesis that grandiose narcissistic traits will relate to greater difficulties in acquiring OC following the inductions (hypothesis ii) was partially supported. Not grandiose narcissistic traits overall, but particularly its exploitativeness–entitlement subcomponent drove the difficulty in increasing compassion for others, especially specific others. Exploitativeness–entitlement traits are seen as the most maladaptive traits within grandiose narcissism (Cai and Luo, 2018), relying heavily on interpersonal manipulative content. Total OC was made up of general and specific OC, underlining exploitativeness–entitlement overall lack of care for others and their resistance to acquiring OC. Furthermore, for those with high exploitativeness–entitlement traits, the type of the induction played a crucial role when targeting the improvement of compassion for someone close to the self. The OC induction increased OC for someone close to a much greater extent than the SC induction and thus may have triggered the need to protect in-group members (Leibowitz, 1997; Cichocka and Cislak, 2020). Therefore, individuals who tend to extort others and have difficulties with interpersonal relationships seem to have difficulty embracing compassion for others but may be able to improve on these factors within their close personal circle but only when OC is directly targeted through therapeutic exercises.

### Vulnerable narcissistic traits and compassion

The hypotheses that vulnerable narcissistic traits would aid in increasing SC following the inductions, in particular after the SC induction (hypothesis iii), was partially supported. The findings showed a general trait effect of oversensitivity to judgment on SC, meaning that regardless of the induction, SC improved more substantially in those with higher oversensitive traits. However, given that vulnerable narcissistic traits correlated negatively with SC scores to begin with, it needs to be considered that this may have contributed to the finding. The oversensitivity to judgment component refers to the traits which are associated with low self-esteem (Rogoza et al., 2018), increased hypersensitivity (Miller et al., 2018), and the need for approval from the outside (Zeigler-Hill et al., 2008). The positive influence subsequently implies that those in greatest need of SC, that is, the ones who suffer from increased oversensitivity to judgment and reported lower SC at the start, are able to benefit from both SC and OC inductions to the largest extent.

Furthermore, the hypothesis that vulnerable narcissistic traits would provide greater difficulties in increasing OC following the inductions (hypothesis iv) was partially supported. The current results suggest an overall deficit in enhancing total OC for individuals with higher global vulnerable narcissistic traits, irrespective of the induction. However, the type of induction is important to consider when aiming to improve compassion for others in those with higher global vulnerable narcissistic and oversensitivity to judgment traits. When the aforementioned traits were higher, the inductions substantially differed in their effectiveness, with the OC induction producing the greatest improvements in OC compared to the SC induction. While egocentrism traits of vulnerable narcissism are devastating to interpersonal relationships due to their self-centered and maladaptive nature, oversensitivity to judgment is characterized by interpersonal insecurities and thus does not inhibit the formation of social relationships *per se*. Nonetheless, given the interaction between the inductions and oversensitivity traits, it seems that oversensitivity to judgment may aid OC improvements when the focus on others is explicitly instructed. Thus, the hypersensitivity associated with oversensitivity to judgment may not only drive the individual’s insecurities but may further be a therapeutic channel for perceiving other people’s struggles.

Lastly, vulnerable narcissistic traits interacted with the inductions for specific OC. Again, the OC induction increased specific OC after the OC induction comparatively more than that after the SC induction for those with higher vulnerable narcissistic traits. Although this mirrors the previous finding, it adds to the picture by displaying that compassion for others may be viewed differently by those with more vulnerable narcissistic traits when it concerns individuals relevant to the self. While the SC induction made individuals with higher vulnerable narcissistic traits seemingly focus only on the self, the OC induction may have additionally triggered the need for outside approval. Considering that OC is a socially approved behavior (Bierhoff, 2005), exhibiting OC may lead individuals with vulnerable narcissistic traits to believe that their close peers will validate this behavior and thus give them the approval they are seeking.

### Implications

Overall, the findings suggest that the magnitude of the increases in compassion depends on individual differences. Grandiose and vulnerable narcissistic traits are both thought of as devastating to intra- and inter-personal wellbeing in the long run (e.g., Campbell et al., 2011; Kaufman et al., 2020; Wirtz and Rigotti, 2020) and thus could present excellent targets for therapeutic compassion exercises. Greatest improvements in SC were observed for individuals with high oversensitivity to judgment traits. Given that this occurred irrespective of the type of induction, it speaks to their potential perceptiveness and need for more SC. Therefore, individuals who generally have a fragile ego or lack self-assurance, and would thus benefit from SC the most, seem to be able to be trained to be more self-compassionate. Future studies should test whether SC exercises could be an efficient therapeutic tool not only for those high in oversensitivity to judgment traits but also potentially for those with other self-defeating traits, such as individuals with attachment anxiety and avoidance or depression (Wei and Ku, 2007).

Moreover, the interactions between the type of induction and narcissistic traits regarding (specific) OC improvements highlight that the approach chosen for improving compassion needs to be carefully considered. While the OC induction was largely more successful at improving OC than the SC induction, this was specifically the case when exploitativeness–entitlement, global vulnerable narcissistic, and oversensitivity to judgment traits were higher. Given this greater success of the OC induction for individuals with these higher narcissistic traits, it should be considered that a lack of compassion for others may be counteracted when OC is directly targeted. Furthermore, following the notion that implementing a communal focus in individuals with increased grandiose narcissistic traits can decrease narcissistic states (Giacomin and Jordan, 2014), and considering that exploitativeness–entitlement is the most devastating of the narcissistic traits to interpersonal relationships, the current results indicate a starting point to further investigate if specifically targeting the increase in OC can decrease destructive behavior of grandiose narcissism toward others, especially close others. Likewise, the current results indicate a potential of oversensitivity to judgment traits to be receptive to other people’s hardship when instructed to focus on others, instead of the self, and pave the way for therapeutic interventions to consider utilizing oversensitivity traits to improve interpersonal relationships.

### Strengths and limitations

Strengths of this study include that both SC and OC were induced and measured according to the empirically supported three-component structure of compassion. Furthermore, this research incorporated the distinction between general and specific OC, which led to delicate and meaningful differences in results. Additionally, the focus on the narcissistic subscales provided for fine-grained analyses. Regardless, some limitations remain. The OC state measures have not been validated previously. The results indicate that they are able to measure state OC well, but future research is needed to confirm this. Moreover, the cross-sectional nature of this experiment limits the assumptions regarding the longevity of the improvements after the inductions. Furthermore, following Weber’s (2007) guidelines, no alpha corrections were applied; thus, the number of analyses conducted may have inflated the possibility of type I error rates. In addition, inductions, especially the OC induction, may be susceptible to demand characteristics, due to the possibility that participants intuit their aim. While the participants who were able to concretely guess the aim of the research were excluded, we are unable to dismiss the possibility of demand characteristics influencing our results. Previous research examined the possible influence of demand characteristics in narcissistic individuals on their results and found that inducing a communal focus (similar to OC) did not alter participants’ responses to appear more socially acceptable (e.g., Giacomin and Jordan, 2014). Given these previous findings and that we found both positive and negative associations between narcissistic traits and compassion, it seems fair to assume that the chance that our results were driven by demand characteristics is slim. However, it would be preferable if future research includes additional measures to detect possible demand characteristics to confirm. Moreover, while the change scores utilized take pre-induction compassion scores into account, future research may aim to consider controlling for baseline compassion traits, in addition to the narcissistic traits, to see if the compassion improvements differ depending on the initial compassionate starting point. Future research should further consider replicating the methods in a larger sample to validate the findings and in individuals with pathological narcissism, potentially using different conceptualisations of narcissism, such as the tripartite model (e.g., Miller et al., 2021) and different narcissism scales, for example, the five-factor narcissism inventory (Glover et al., 2012) or the pathological narcissism inventory (Pincus et al., 2009).

## Conclusion

Generally, narcissistic traits (particularly grandiose traits) were expected to aid in increasing SC and hindering increases in OC (especially general OC). The current results indicate that vulnerable narcissistic oversensitivity to judgment traits relate to improvements in SC following the inductions. Moreover, grandiose exploitativeness–entitlement and global vulnerable narcissistic traits evidently impair the overall capacity to increase compassion for others. Nonetheless, when exploitativeness–entitlement, global vulnerable narcissistic, and oversensitivity to judgment traits were higher, inducing compassion for others resulted in greatest improvements in OC compared to inducing compassion for the self. Overall, given the superior effectiveness of one induction over the other when narcissistic traits were high, the current results suggest that other compassionate behavior could be specifically trained when targeted directly, even in individuals who show a general resistance to acquiring OC. Moreover, the theorized lack of SC in individuals with vulnerable oversensitivity to judgment traits seems possible to be counteracted through different types of compassion exercises.

## Data availability statement

The datasets presented in this study can be found in online repositories. The names of the repository/repositories and accession number(s) can be found below: https://osf.io/6e2vz/?view_only=74acc5572d2a4f958f753eea991613ba.

## Ethics statement

The studies involving human participants were reviewed and approved by Ethics Review Committee Psychology and Neuroscience, Maastricht University. The patients/participants provided their written informed consent to participate in this study.

## Author contributions

All authors contributed to the study conception and design. VF wrote the first draft of the manuscript. All authors have read and approved the final manuscript.

## Footnotes

1 Freund et al. (under review). Self - And Other-Compassion’s Malleability: Can Compassion Be Induced And Who Benefits?. Available Online at: https://mfr.de-1.osf.io/render?url=https://osf.io/b57gy/?direct%26mode=render%26action=download%26mode=render.

2 The original pre-registration was formulated for a larger project and summarized hypotheses for the current paper (narcissistic traits) and hypotheses regarding the influence of other personality traits. These hypotheses were disentangled to promote comprehensibility of the individual results.

3
https://osf.io/ue9jf/?view_only=8c41cb4be3154f11be866dfbae6754cd

4
https://osf.io/6e2vz/?view_only=74acc5572d2a4f958f753eea991613ba

5 The original analysis plan aimed to analyze the results using ANCOVAS, however, given that the independence of residuals is violated when using repeated measures (i.e., across timepoints; pre- and post-induction scores), and that ANCOVAs assume that the interaction effect is non-existent (see e.g., Montoya, 2019), which is contrary to our hypotheses, the current study employed moderation regression analyses.
