# Supplementary material for: Narcissistic traits and compassion: Embracing oneself while devoiding others
Source: Front Psychol. 2022 Oct 11;13:914270. doi: 10.3389/fpsyg.2022.914270 (PMC9592718; doi:10.3389/fpsyg.2022.914270)
Supplement: Supplementary file 1 [file Table_1.docx]

**Appendix 1.**

State Other-Compassion Scale

Please think about a situation someone you know is experiencing right now that is painful or difficult. It could be some challenge in their life, or perhaps they are feeling inadequate in some way. Please indicate how well each statement applies to how you are **feeling toward other people in GENERAL right now.**

1. Right now, I’m caring toward people who are going through a difficult time.
2. Right now, I’m being there for others in times of difficulty.
3. Right now, my heart is going out to people who are unhappy.
4. Right now, I’m comforting people who feel sadness.
5. Right now, I’m remembering that everyone feels down sometimes; it is part of being human.
6. Right now, I’m recognizing that all people have weaknesses and no one’s perfect.
7. Right now, I know that everyone feels pain just like me.
8. Right now, I’m aware that suffering is just a part of the common human experience.
9. Right now, I’m paying careful attention when other people talk to me.
10. Right now, I’m noticing when people are upset, even if they don’t say anything.
11. Right now, I listen patiently to other people’s problems.
12. Right now, I’m keeping a balanced perspective on problems people tell me about.
13. Right now, I’m not concerning myself with other people’s problems.
14. Right now, I’m not connecting with other people who are suffering.
15. Right now, I’m not thinking much about the concerns of others.
16. Right now, I’m avoiding people who are experiencing a lot of pain.
