# Supplementary material for: Narcissistic traits and compassion: Embracing oneself while devoiding others
Source: Front Psychol. 2022 Oct 11;13:914270. doi: 10.3389/fpsyg.2022.914270 (PMC9592718; doi:10.3389/fpsyg.2022.914270)
Supplement: Supplementary file 2 [file Table_2.docx]

**Appendix 2.**

State Specific-Other-Compassion Scale

Please think about a situation that someone you know is experiencing right now that is painful or difficult. It could be some challenge in their life, or perhaps they are feeling inadequate in some way. Please indicate how well each statement applies to how you are **feeling toward that SPECIFIC person right now** as you think about their situation.

*(Participants were asked to identify a specific person to think of while completing the questions, the specific person’s name was automatically inserted into the questions below.)*

1. Right now, I feel caring toward ...
2. Right now, I am there for...
3. Right now, my heart is going out to …
4. Right now, I’m comforting …
5. Right now, I’m remembering that … feels down sometimes; it is part of being human.
6. Right now, I’m recognizing that … has weaknesses and no one’s perfect.
7. Right now, I know that … feels pain just like me.
8. Right now, I’m aware that … suffering is just a part of the common human experience.
9. Right now, I pay careful attention when … talks to me.
10. Right now, I notice when … is upset, even if … doesn’t say anything.
11. Right now, I listen patiently to …’s problems.
12. Right now, I’m keeping a balanced perspective on problems … tells me about.
13. Right now, I’m not concerning myself with …’s problems.
14. Right now, I’m not connecting with … who is suffering.
15. Right now, I’m not thinking much about the concerns of ...
16. Right now, I’m avoiding … who is experiencing a lot of pain.
