# Supplementary material for: Narcissistic traits and compassion: Embracing oneself while devoiding others
Source: Front Psychol. 2022 Oct 11;13:914270. doi: 10.3389/fpsyg.2022.914270 (PMC9592718; doi:10.3389/fpsyg.2022.914270)
Supplement: Supplementary file 3 [file Table_3.docx]

**Appendix 3.**

Other-Compassion Induction

“Please think about a particular situation someone you know is experiencing right now that is painful or difficult. It could be some struggle in their life, or perhaps they are feeling inadequate in some way. Please think of a situation which doesn’t involve you directly. Decide on a single situation that you will focus on throughout this study.”

*“We would now like you to take part in a brief exercise, to see if it is helping you relate to this person’s struggle.”*

1. **Mindfulness Writing Prompt**

Please complete this brief writing exercise and follow the instructions as closely as possible.

In space below, please write about what thoughts and emotions might be coming up for you as you think about this other person's difficult situation. See if you can allow yourself to open to this person's pain, to be with it with a spirit of courage and openheartedness.

For example, “I feel so badly that my friend is struggling after her break-up. I know how hard it is for her to be going through this. (…)*”*

Remember-- your responses are completely anonymous, and your writing is confidential. Don’t worry about spelling, sentence structure, or grammar.

1. **Common Humanity Writing Prompt**

In the space below, please write about how people may share similar feelings when encountering situations like this.

Consider that experiencing difficult situations is a part of being human. Although the way people struggle is different and the amount of challenge varies, all people face difficulties in life. What they are experiencing is not abnormal but is a part of life.

For example, “I guess everyone gets their heart broken sometimes. It's part of being human. (…)”

Remember-- your responses are completely anonymous, and your writing is confidential. Don’t worry about spelling, sentence structure, or grammar.

1. **Kindness Writing Prompt**

In the space below, please get in touch with any feelings of kindness, care, understanding or concern for this person as they are going through this difficulty. Imagine that you are writing to this person. What words of support, encouragement and kindness would you like to express?

For example, “*You're doing the best you can. I'm so sorry you're struggling with this. It's going to be okay. I will help you and support you to get through this. (…)”*

Remember-- your responses are completely anonymous, and your writing is confidential. Don’t worry about spelling, sentence structure, or grammar.

Please take some time to read what you wrote to them and see how it feels to give these words of kindness and concern. Imagine how it feels for the other person to hear these words of kindness and concern. Notice if anything could be particularly comforting or helpful to the other person. Take a few slow, deep breaths as you read what you’ve written.
