# Supplementary material for: Narcissistic traits and compassion: Embracing oneself while devoiding others
Source: Front Psychol. 2022 Oct 11;13:914270. doi: 10.3389/fpsyg.2022.914270 (PMC9592718; doi:10.3389/fpsyg.2022.914270)
Supplement: Supplementary file 4 [file Table_4.docx]

**Appendix 4**

|  | 1 | 2 | 3 | 4 | | | 5 | | 6 | 7 | 8 | 9 | 10 | 11 | 12 | 13 | 14 | 15 | 16 |
| --- | --- | --- | --- | --- | --- | --- | --- | --- | --- | --- | --- | --- | --- | --- | --- | --- | --- | --- | --- |
| Grandiose narcissistic traits |  |  |  |  | | |  | |  |  |  |  |  |  |  |  |  |  |  |
| 1. Global | - |  |  |  | | |  | |  |  |  |  |  |  |  |  |  |  |  |
| 2. Leadership-authority | .77*** | - | | |  |  | |  |  |  |  |  |  |  |  |  |  |  |  |
| 3. Self-absorption –  self- administration | .71*** | .33*** | - |  | | |  | |  |  |  |  |  |  |  |  |  |  |  |
| 4. Superiority-arrogance | .86*** | .62*** | .47*** | - | | |  | |  |  |  |  |  |  |  |  |  |  |  |
| 5. Exploitativeness-entitlement | .72*** | .32*** | .41*** | .53*** | | | - | |  |  |  |  |  |  |  |  |  |  |  |
| Vulnerable narcissistic traits |  |  |  |  | | |  | |  |  |  |  |  |  |  |  |  |  |  |
| 6. Global | .18** | -.13* | .10 | .15* | | | .49*** | | - |  |  |  |  |  |  |  |  |  |  |
| 7. Oversensitivity to judgement | .11 | -.16* | .08 | 08 | | | .41*** | | .91*** | - |  |  |  |  |  |  |  |  |  |
| 8. Egocentrism | .19** | -.07 | .11 | .16* | | | .45*** | | .81*** | .54*** | - |  |  |  |  |  |  |  |  |
| Compassion |  |  |  |  | | |  | |  |  |  |  |  |  |  |  |  |  |  |
| Change scores |  |  |  |  | | |  | |  |  |  |  |  |  |  |  |  |  |  |
| 9. Self-compassion | -.02 | -.12 | .10 | -.05 | | | .05 | | .18** | .17** | .16* | - |  |  |  |  |  |  |  |
| 10. Total other-compassion | -.15* | -.18** | -.07 | -.14* | | | -.06 | | .06 | .09 | .04 | .17** | - |  |  |  |  |  |  |
| 11. General other-compassion | -.18** | -.17** | -.10 | -.17* | | | -.09 | | .002 | .05 | -.03 | .15* | .89*** | - |  |  |  |  |  |
| 12. Specific other-compassion | -.06 | -.13 | .003 | -.05 | | | .02 | | .13 | .11 | .13* | .14* | .75*** | .35*** | - |  |  |  |  |
| Pre-induction scores |  |  |  |  | | |  | |  |  |  |  |  |  |  |  |  |  |  |
| 13. Self-compassion | -.01 | .24*** | -.02 | .01 | | | -.29*** | | -.49*** | -.49*** | -.34*** | -.53*** | -.03 | -.04 | -.01 | - |  |  |  |
| 14. Total other-compassion | -.01 | .08 | .09 | -.07 | | | -.16* | | -.26*** | -.13* | -.32*** | -.002 | -.28*** | -.23*** | -.23*** | .21** | - |  |  |
| 15. General other-compassion | -.01 | .05 | .07 | -.12 | | | -.21** | | -.28*** | -.13* | -.36*** | .02 | -.25*** | -.31*** | -.05 | .24*** | .90*** | - |  |
| 16. Specific other-compassion | .04 | .08 | .09 | .01 | | | -.07 | | -.17* | -.11 | -.19** | -.02 | -.25*** | -.09 | -.38*** | .14* | .87*** | .56*** | - |

*Pearson correlations between study variables; grandiose and vulnerable narcissistic trait and compassion change scores and pre-induction scores.*

*Note*: **p* < .05; ***p* < .01; ****p* < .0001.
