# Supplementary material for: Narcissistic traits and compassion: Embracing oneself while devoiding others
Source: Front Psychol. 2022 Oct 11;13:914270. doi: 10.3389/fpsyg.2022.914270 (PMC9592718; doi:10.3389/fpsyg.2022.914270)
Supplement: Supplementary file 5 [file Table_5.docx]

**Appendix 5**

*Dependant t-tests between pre- and post-induction compassion scores.*

|  | *M_dif_* | *SD* | *t* | *df* | *p* |
| --- | --- | --- | --- | --- | --- |
| Compassion scores pre vs post |  |  |  |  |  |
| SC | .30 | .52 | 8.79* | 229 | <.001 |
| Total OC | .13 | .32 | 6.19* | 229 | <.001 |
| General OC | .13 | .45 | 4.35* | 229 | <.001 |
| Specific OC | .13 | .32 | 6.25* | 229 | <.001 |

*Note*: * *p* < .001. Positive values indicate an increase of compassion scores after the inductions.
