# Supplementary material for: Narcissistic traits and compassion: Embracing oneself while devoiding others
Source: Front Psychol. 2022 Oct 11;13:914270. doi: 10.3389/fpsyg.2022.914270 (PMC9592718; doi:10.3389/fpsyg.2022.914270)
Supplement: Supplementary file 6 [file Table_6.docx]

**Appendix 6**

*Moderation regression analyses for grandiose narcissistic traits.*

| Change | Model | Induction | | Trait | | Interaction: induction x trait | | | | Effect size |
| --- | --- | --- | --- | --- | --- | --- | --- | --- | --- | --- |
| State | # | *t* | *p* | *t* | *p* | Score | *t* | | *p* | *R^2^* |
| SC | 1 |  |  | Global | |  | | | |  |
|  |  | -4.86* | <.0001 | .93 | .35 |  | -1.08 | .28 | | .10 |
|  | 2 |  |  | Leadership-Authority | |  | | | |  |
|  |  | -4.88* | <.0001 | -.63 | .53 |  | .08 | .94 | | .11 |
|  | 3 |  |  | Self-administration-Self-absorption | |  |  |  | |  |
|  |  | -4.78* | <.0001 | 1.63 | .11 |  | -1.53 | .13 | | .11 |
|  | 4 |  |  | Superiority-Arrogance | |  |  |  | |  |
|  |  | -4.80* | <.0001 | .46 | .65 |  | -.69 | .49 | | .10 |
|  | 5 |  |  | Exploitativeness-Entitlement | |  |  |  | |  |
|  |  | -5.05* | <.0001 | 1.69 | .09 |  | -1.57 | .12 | | .12 |
| OC | 6 |  |  | Global | |  | | | |  |
|  |  | 2.60* | .01 | -.74 | .46 |  | .05 | .96 | | .05 |
|  | 7 |  |  | Leadership-Authority | |  | | | |  |
|  |  | 2.51* | .01 | .15 | .88 |  | -1.01 | .31 | | .07 |
|  | 8 |  |  | Self-administration-Self-absorption | |  |  |  | |  |
|  |  | 2.31* | .02 | .27 | .79 |  | -.53 | .60 | | .03 |
|  | 9 |  |  | Superiority-Arrogance | |  |  |  | |  |
|  |  | 2.66* | .008 | -.85 | .39 |  | .17 | .87 | | .05 |
|  | 10 |  |  | Exploitativeness-Entitlement | |  |  |  | |  |
|  |  | 2.66* | .009 | -2.05* | .04 |  | 1.51 | .13 | | .04 |
| general OC | 11 |  |  | Global | |  | | | |  |
|  |  | 2.85* | .005 | -.66 | .51 |  | -.18 | .86 | | .07 |
|  | 12 |  |  | Leadership-Authority | |  | | | |  |
|  |  | 2.74* | .007 | -.23 | .82 |  | -.57 | .57 | | .06 |
|  | 13 |  |  | Self-administration-Self-Absorption | |  |  |  | |  |
|  |  | 2.53* | .01 | .58 | .56 |  | -.97 | .33 | | .04 |
|  | 14 |  |  | Superiority-Arrogance | |  |  |  | |  |
|  |  | 2.92* | .004 | -.70 | .48 |  | -.15 | .88 | | .06 |
|  | 15 |  |  | Exploitativeness-Entitlement | |  |  |  | |  |
|  |  | 3.00* | .003 | -1.40 | .16 |  | .72 | .48 | | .05 |
| specific OC | 16 |  |  | Global | |  | | | |  |
|  |  | 1.14 | .26 | -.58 | .56 |  | .36 | .72 | | .01 |
|  | 17 |  |  | Leadership-Authority | |  | | | |  |
|  |  | 1.12 | .27 | .62 | .54 |  | -1.30 | .20 | | .03 |
|  | 18 |  |  | Self-administration-Self-Absorption | |  |  |  | |  |
|  |  | 1.06 | .30 | -.19 | .85 |  | .24 | .81 | | .01 |
|  | 19 |  |  | Superiority-Arrogance | |  |  |  | |  |
|  |  | 1.17 | .25 | -.75 | .46 |  | .56 | .58 | | .01 |
|  | 20 |  |  | Exploitativeness-Entitlement | |  |  |  | |  |
|  |  | 1.07 | .29 | -1.91 | .06 |  | 2.02* | .04 | | .02 |
|  |  |  |  |  |  | Low | -.57 | .57 | |  |
|  |  |  |  |  |  | High | 2.08* | .04 | |  |

*Note: * p < .05.*
