# Supplementary material for: Narcissistic traits and compassion: Embracing oneself while devoiding others
Source: Front Psychol. 2022 Oct 11;13:914270. doi: 10.3389/fpsyg.2022.914270 (PMC9592718; doi:10.3389/fpsyg.2022.914270)
Supplement: Supplementary file 7 [file Table_7.docx]

| Change | Model | Induction | | Trait | | Interaction: induction x trait | | | | Effect size |
| --- | --- | --- | --- | --- | --- | --- | --- | --- | --- | --- |
| State | # | *t* | *p* | *t* | *p* | Score | *t* | | *p* | *R^2^* |
| SC | 21 |  |  | Global | |  | | | |  |
|  |  | -5.40* | <.0001 | 1.81 | .07 |  | -.92 | .36 | | .15 |
|  | 22 |  |  | Oversensitivity to Judgment | |  | | | |  |
|  |  | -5.42* | <.0001 | 2.33* | .02 |  | -1.58 | .12 | | .15 |
|  | 23 |  |  | Egocentrism | |  |  |  | |  |
|  |  | -5.16* | <.0001 | .66 | .51 |  | .18 | .86 | | .13 |
| OC | 24 |  |  | Global | |  | | | |  |
|  |  | 2.37* | .02 | -2.08* | .04 |  | 2.47* | .01 | | .05 |
|  |  |  |  |  |  | Low | .09 | .93 | |  |
|  |  |  |  |  |  | High | 2.99* | .003 | |  |
|  | 25 |  |  | Oversensitivity to Judgment | |  | | | |  |
|  |  | 2.31* | .02 | -1.70 | .09 |  | 2.15* | .03 | | .05 |
|  |  |  |  |  |  | Low | .38 | .71 | |  |
|  |  |  |  |  |  | High | 2.90* | .004 | |  |
|  | 26 |  |  | Egocentrism | |  |  |  | |  |
|  |  | 2.43* | .02 | -1.66 | .10 |  | 1.71 | .09 | | .04 |
| general OC | 27 |  |  | Global | |  | | | |  |
|  |  | 2.71* | .007 | -1.64 | .10 |  | 1.70 | .09 | | .05 |
|  | 28 |  |  | Oversensitivity to Judgment | |  | | | |  |
|  |  | 2.61* | .01 | -1.33 | .18 |  | 1.54 | .13 | | .04 |
|  | 29 |  |  | Egocentrism | |  |  |  | |  |
|  |  | 2.77* | .006 | -1.45 | .15 |  | 1.21 | .23 | | .04 |
| specific OC | 30 |  |  | Global | |  | | | |  |
|  |  | .89 | .38 | -1.60 | .11 |  | 2.35* | .02 | | .04 |
|  |  |  |  |  |  | Low | -.93 | .35 | |  |
|  |  |  |  |  |  | High | 2.11* | .04 | |  |
|  | 31 |  |  | Oversensitivity to Judgment | |  | | | |  |
|  |  | .92 | .36 | -1.25 | .21 |  | 1.86 | .06 | | .03 |
|  | 32 |  |  | Egocentrism | |  |  |  | |  |
|  |  | .94 | .35 | -1.21 | .23 |  | 1.96 | .05 | | .04 |
|  |  |  |  |  |  | Low | -.61 | .55 | |  |
|  |  |  |  |  |  | High | 1.89 | .06 | |  |

**Appendix 7**

*Moderation regression analyses for vulnerable narcissistic traits.*

*Note. * p < .05.*
